# Geochemical Characterization of the NWA 11273 Lunar Meteorite Using Nondestructive Analytical Techniques: Original, Shocked, and Alteration Mineral Phases

**DOI:** 10.1021/acsearthspacechem.0c00329

**Published:** 2021-05-27

**Authors:** Jennifer Huidobro, Julene Aramendia, Gorka Arana, Juan Manuel Madariaga

**Affiliations:** Analytical Chemistry Department, University of the Basque Country (UPV/EHU), Barrio Sarriena s/n, 48980 Leioa, Spain

**Keywords:** Raman spectroscopy, X-ray spectroscopy, nondestructive analytical techniques, Raman imaging, lunar meteorite, shocked zircon, terrestrial weathering, chemical alteration

## Abstract

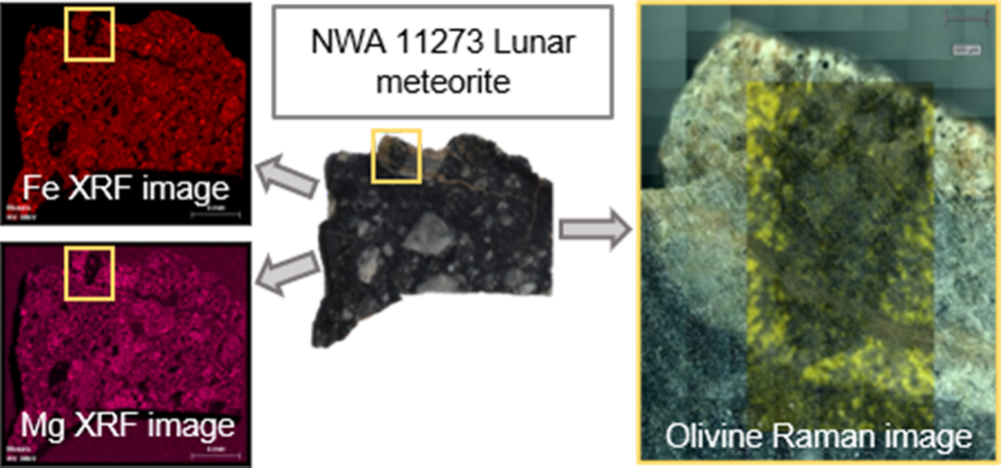

A lunar feldspathic breccia meteorite, the Northwest Africa (NWA) 11273, was analyzed to compensate the lack of scientific data available about its mineralogy and geochemistry. In order to obtain a deeper characterization of the sample, a strategy based on the combination of nondestructive spectroscopic techniques such as X-ray fluorescence and Raman spectroscopy is used. Both techniques are being used in spatial missions by the Perseverance Rover, so their combination in the laboratory is here proposed as an optimal strategy to study the complete mineralogy of the sample. In addition to finding the minerals indicated by the Meteoritical Society (anorthite, olivine, pyroxene, kamacite, and troilite), other minor minerals were identified, such as zircon and ilmenite, which are minerals related to the Moon geology, as well as calcite and sulfate which can be considered products of terrestrial weathering. Finally, secondary minerals related to alteration processes were also found, such as hematite, quartz, and anatase. In this work, the alteration processes that gave rise to the detected secondary minerals have been proposed.

Space is being continuously explored with the aim of answering fundamental issues about the origin and the history of the Solar System. Moreover, remarkable advances and innovative technology design are necessary to understand better the universe and to predict the evolution of the Solar System in the future, providing a great progress in science and technology.

While remote and contact investigations of planets, asteroids, and other objects that orbit the Sun are essential for understanding planetary processes, much of the geochemical and geological information regarding the Solar System comes directly from the study of rocks and other materials originating from them.^1^ In this way, meteorites are fundamental scientific materials, providing information on past conditions within planets, and on their surfaces, and revealing the timing of key events that affected a planet’s evolution.^1^

Most meteorites belong to the asteroids belt, although a few rare meteorites found on Earth have chemical signatures that suggest they come from Mars or the Earth’s Moon.^2^ Lunar and Martian meteorites were formed due to random impact events on the body’s surface, ejecting the loosened material into space.^3^ In the case of Lunar meteorites, they can provide information about areas of the Moon not sampled by the Apollo and Luna missions, which collected samples from a relatively small and geochemically anomalous region of the surface,^4^ and they potentially offer a broad insight into the petrology, geochemistry, and geochronology of the lunar crust.^3^

The Moon surface is differentiated by two distinct areas: the regolith and the lunar marias. Almost the entire Moon is covered by the lunar regolith that is a light-colored dust and rocky rubble. Conversely, the marias are craters filled by dark materials that belong to the Moon core.

The study of nonterrestrial materials is crucial because they also may act as historical tracers, allowing us to deduce the composition of the body’s origin, temperature, and pressure reached during the impact, signs of water presence, and more.^5^ The mineral phases belonging to the parent body are known as primary minerals. However, primary minerals that have been altered by pressure, temperature, or other factors are known as secondary minerals.^6^ Mineral phases that are neither primary nor secondary can be found when studying a nonterrestrial sample. These mineral phases may come from the terrestrial weathering, for example, the entrance of water through cracks and the subsequent precipitation of compounds, the biological activity of organisms, and more. Nowadays, analytical methods allow performing in-depth analysis in order to identify the elemental and molecular composition of these samples.

To perform the geological and mineralogical characterization of nonterrestrial samples, several analytical techniques can be used, such as inductively coupled plasma–mass spectrometry (ICP–MS)^7^ and inductively coupled plasma–atomic emission spectroscopy (ICP–AES)^5^ in the field of elemental characterization; infrared spectroscopy (IR)^8^ in the determination of hydrated mineral forms; or, for example, X-ray diffraction (XRD)^9^ to extract the mineralogical and structural information of the sample.^5^ When analyzing rare materials (as is the case of meteorites), the main drawback of the mentioned techniques is that they require sample preparation and/or destruction. However, each of the nonterrestrial samples is unique and special, so it is necessary to preserve its integrity as much as possible. For this reason, the so-called nondestructive or noninvasive analytical techniques are increasingly used.

Particle-induced X-ray emission (PIXE),^10^ micro-PIXE,^10^ secondary ion mass spectrometry (SIMS),^11^ nano-SIMS,^12^ X-ray fluorescence spectroscopy (XRF),^13^ scanning electron microscopy–energy-dispersive X-ray spectroscopy (SEM–EDS),^14^ and electron microprobe analyzer (EPMA)^15^ are the most common noninvasive techniques used in the elemental characterization of extraterrestrial samples. In the case of the molecular study of these material, Mössbauer spectroscopy,^16^ IR, and Raman spectroscopy^17^ are the most common nondestructive analytical techniques to study the molecular composition of these samples.

Considering the increasing importance that the spectroscopic techniques are acquiring in the field of the planetary exploration (e.g., Sherloc, PIXL, SuperCam, RLS, and MicroOmega onboard the Mars2020 and ExoMars rovers), in this work, we employ spectroscopic laboratory techniques to carry out an exhaustive elemental and molecular characterization of a sample of high relevance for planetary mission.

In detail, this work focuses on the study of the lunar meteorite NWA 11273, which has been classified as a feldspar breccia, achondrite, by the Meteoritical Society.^18^ According to the petrographic analysis provided by the Meteoritical Bulletin, the meteorite is composed of anorthite [CaAl_2_Si_2_O_8_],^19^ olivine [(Mg,Fe)_2_SiO_4_],^20^ clinopyroxene, and orthopyroxene [(Ca,Mg,Fe,Mn,Na,Li) (Al,Mg,Fe,Mn,Cr,Sc,Ti) (Si,Al)_2_O_6_],^20^ pigeonite [(Mg,Fe,Ca)SiO_3_],^21^ exsolved pigeonite, chromite [FeCr_2_O_4_],^22^ spinel [(Mg,Fe)(Al,Fe,Cr)_3_O_4_],^23^ kamacite [α(Fe,Ni)],^24^ taenite [γ(Fe,Ni)],^24^ and troilite [FeS]^25^ in a finer grained matrix containing small vesicles and minor barite [BaSO_4_].^26^

As, by the moment, there are not more scientific publications about the mineralogy of the NWA 11273 Lunar meteorite, the aim of this work is to make a deeper characterization of this meteorite through nondestructive techniques that are currently operating on Mars. Therefore, by obtaining high-quality and high-resolution analyses, minority mineral phases, apart from the majority ones, can be found. Knowing the minority minerals of a planet or satellite is of great importance, since these small compounds allow us to determine alteration processes or processes of the formation of other secondary minerals. These are the elemental keys for reconstructing the history of the meteorite and the geological unit from which they originate.

To do so, two nondestructive analytical techniques have been selected. These are XRF and Raman spectroscopy. XRF was selected because this technique was one of the pioneers in travelling into space under the name RIFMA XRF spectrometer in the Luna 17 mission which was launched in 1970. In addition, XRF has been used as a standard analytical method in space and Earth science.^27,28^

Although Raman spectroscopy is widely used to assess the molecular (mineralogical) composition of terrestrial materials in the laboratory, its application in space exploration missions is just at the beginning. In detail, the NASA’s Perseverance Rover that landed at Jezero crater on February 18th 2021 is equipped with two Raman instruments, named SuperCam^29^ and Sherloc,^30^ and the analytical payload selected for the Rosalind Franklin Rover planned to land at Oxia Planum in 2023 is equipped with the so-called Raman laser spectrometer (RLS).^31^ Focusing on current missions, it is important to underline that the Sherloc instrument onboard the Mars2020 Rover will operate in coordination with the Planetary Instrument for X-ray Lithochemistry (PIXL),^32^ which is an X-ray spectrometer that will map the elemental composition of selected superficial targets. As the combination of nondestructive XRF and Raman measurements has been selected by NASA as a recommended strategy to determine the mineralogical and geochemical composition of Martian targets, this paper also aims at providing valuable clues about the potential scientific outcome that could be derived from this approach when analyzing extraterrestrial materials.

## Experimental Section

### Sample

For this work, a thin sample of the Northwest Africa (NWA) 11273 Lunar meteorite (Figure S1 Supporting Information), acquired by the IBeA research group, was analyzed. This meteorite was found in April 2017 near Tindouf, Algeria. It had a total mass of 2.81 kg, and a specimen of 37 g was sent to the University of Washington (UWS) to be analyzed. Finally, the NWA 11273 was classified as a lunar feldspar breccia meteorite on October 2017 with a low shock stage and low weathering grade^18^ by the Meteoritical Society.

The analyzed sample is a thin section, whose dimensions are 2.1 × 1.9 cm, with a weight of 0.61 g (0.02% of the original recovered meteorite mass) and it shows heterogeneous fragments of light-gray color embedded in a darker color section. This description corresponds to the meaning of a breccia.^33^

### Instrumentation

#### Micro-X-ray Fluorescence Spectrometer

The M4 TORNADO micro-energy-dispersive X-ray fluorescence (m-ED-XRF) spectrometer was used with the aim of investigating the elemental composition and the element distribution in the studied sample. It can detect elements with the atomic number (*Z*) higher than 10 (starting from sodium). It implements two Rh tubes powered by a low-power high-voltage (HV) generator and cooled by air. One of the tubes is able to operate at voltages in the range of 10–50 kV and currents in the range of 100–700 mA. This first tube is mounted on a mechanical collimator that allows performing measurements under a lateral/spatial resolution (spot) of 1 mm. There is a second Rh tube which can operate between 10–50 kV and 100–600 mA. This polycapillary lens is able to achieve a lateral resolution of 25 μm for the Mo Kα-line. The detection of the fluorescence radiation emitted by the elements is performed by an energy-dispersive SDD detector with 30 mm^2^ sensitive area and an energy resolution of 142 eV for the Mn Kα line. The system can work under vacuum conditions in order to improve the detection of lighter elements (*Z* < 16). For that purpose, an MV 10 N VARIO-B diaphragm pump was used establishing the vacuum inside the chamber of the instrument at 20 mbar.

#### Micro-Raman Spectroscopy

Micro-Raman spectroscopy was used to obtain the molecular (mineralogical) composition by measuring the sample surface. Different Raman spectrometers were employed with different aims, the Renishaw inVia and the Renishaw RA-100.

The inVia Raman microspectrometer (Renishaw, UK) is equipped with 785 and 532 nm excitation diode lasers and with a CCD detector cooled by the Peltier effect.The nominal power of the source can be modulated between 0.0001 and 100% of the total power to avoid thermodecomposition of the sample. This spectrometer was employed to perform Raman imaging, which helped us to define the spatial distribution of the compounds in the meteorite bulk. The spectral resolution was about 1 cm^–1^ (indicated by the manufacturer) and the used objectives were 5×, 20×, and 50×. The spectra were obtained in a range of 100–1800 or 100–3200 cm^–1^, depending on the aim of the analysis.

Raman images were obtained using the high-resolution stream line setup (Renishaw, UK) coupled to the inVia spectrometer. The control software moves the sample beneath the lens of the inVia’s motorized microscope stage, so the line is rastered across the region of interest with a spatial resolution up to 1 μm and moving in the snake mapping mode, which involves movement from the right end of the top row to the right end of the next row. At the end, 2D Raman images are obtained containing individual Raman information at the pixel level.

To carry out some punctual analysis, the microprobe Renishaw RA-100 Raman spectrometer (Renishaw, UK) was used. It is equipped with a 785 nm excitation diode laser and with a CCD detector cooled by the Peltier effect. In this case, the nominal power at the source was about 150 mW and some filters allowed working with 1, 10, or 100% of the total laser power to avoid the transformation of the sample due to the high temperatures. The spectra were obtained in a range of 200–2200 cm^–1^ with a spectral resolution of 2 cm^–1^ and 20× and 50× objectives.

In both cases, the spectrometers were calibrated setting the 520 cm^–1^ band (corresponding to silicon) and the number of accumulations and the acquisition time were optimized for each measurement in order to improve the signal-to-noise ratio. The software used for data analysis was the Wire 4.2 (Renishaw, UK) for the inVia Raman and the Wire3.2 (Renishaw, UK) for the RA-100 Raman. Finally, public databases such as RRUFF^34^ and bibliography were checked in order to interpret the spectra.

## Results and Discussion

A general analysis of the surface was performed by means of micro-XRF imaging for assessing the elemental composition of the whole surface of the sample by the two sides (Supporting Information S1). Combining these element mappings, some hotspots where different elements coexisted were detected. Afterward, Raman analyses were carried out in those areas to identify the mixture of minerals the meteorite was composed of.

According to XRF and Raman analysis, the most-abundant discrete mineral imbedded in the feldspathic breccia family of meteorites belongs to the plagioclase group,^4^ which is a solid solution ranging from pure albite [NaAlSi_3_O_8_]^35^ to pure anorthite [CaAl_2_Si_2_O_8_].^35^ The peaks of the anorthite found appeared at 281, 487, and 505 cm^–1^,^36,37^ which correspond to the single band around 280 cm^–1^ and a doublet peak between 480 and 508 cm^–1^^38^ that make the common plagioclase Raman spectrum. This compound was detected as the main mineral phase of the NWA 11273 Lunar meteorite.

The second major mineral family found was the pyroxene, which is divided into two subfamilies: orthopyroxene and clinopyroxene. On the one hand, orthopyroxenes crystallize in the orthorhombic system^39^ and its chemical composition ranges from pure magnesium silicate, enstatite [MgSiO_3_],^40^ to pure ferrous iron silicate, ferrosilite [FeSiO_3_].^41^ On the other hand, clinopyroxenes crystallize in the monoclinic system^39^ and its chemical formula is composed by single chains of silica tetrahedra SiO_4_ shared with a large variety of cations. Even though the pyroxene family has a Raman spectrum pattern, which consist of bands in the 300–400, 650–700, and 980–1020 wavenumber region, both subfamilies can be differentiated by their own Raman bands. Orthopyroxenes have a doublet in the 650–700 cm^–1^ region, whereas clinopyroxenes show only one intense band in that region.^42^ In this way, two types of spectra were found. The peaks of the first type of the spectrum appear at 296, 330, 363, 391, 660, 678, and 1001 cm^–1^, whereas the peaks of the second one appear at 323, 355, 389, 666, and 1010 cm^–1^. According to the bases proposed above, the first spectrum is an orthopyroxene because it has a doublet in the 650–700 cm^–1^ region and the second one is a clinopyroxene because it only has one band in that region. However, thanks to the flow chart published by Wang et al.,^43^ the space group of both compounds can be known. On the one hand, as the first spectrum possessed a doublet in the 600–800 cm^–1^ region (660 and 678 cm^–1^), a triplet in the 300–450 cm^–1^ region (330, 363, and 391 cm^–1^), and a band in the 230–300 cm^–1^ region (296 cm^–1^), the compound found is a high magnesium *Pbca* pyroxene. On the other hand, as the second spectrum only has one peak between 600 and 800 cm^–1^ (666 cm^–1^, at a wavenumber higher than the limit of 660 cm^–1^), the compound may be a clinopyroxene or an iron-pyroxenoid. However, as this spectrum has three peaks in the 300–450 cm^–1^ region (323, 355, and 389 cm^–1^) and no peaks below 300 cm^–1^, the compound found is a *C*2_1_/*c* monoclinic pyroxene.

**Figure 1:**
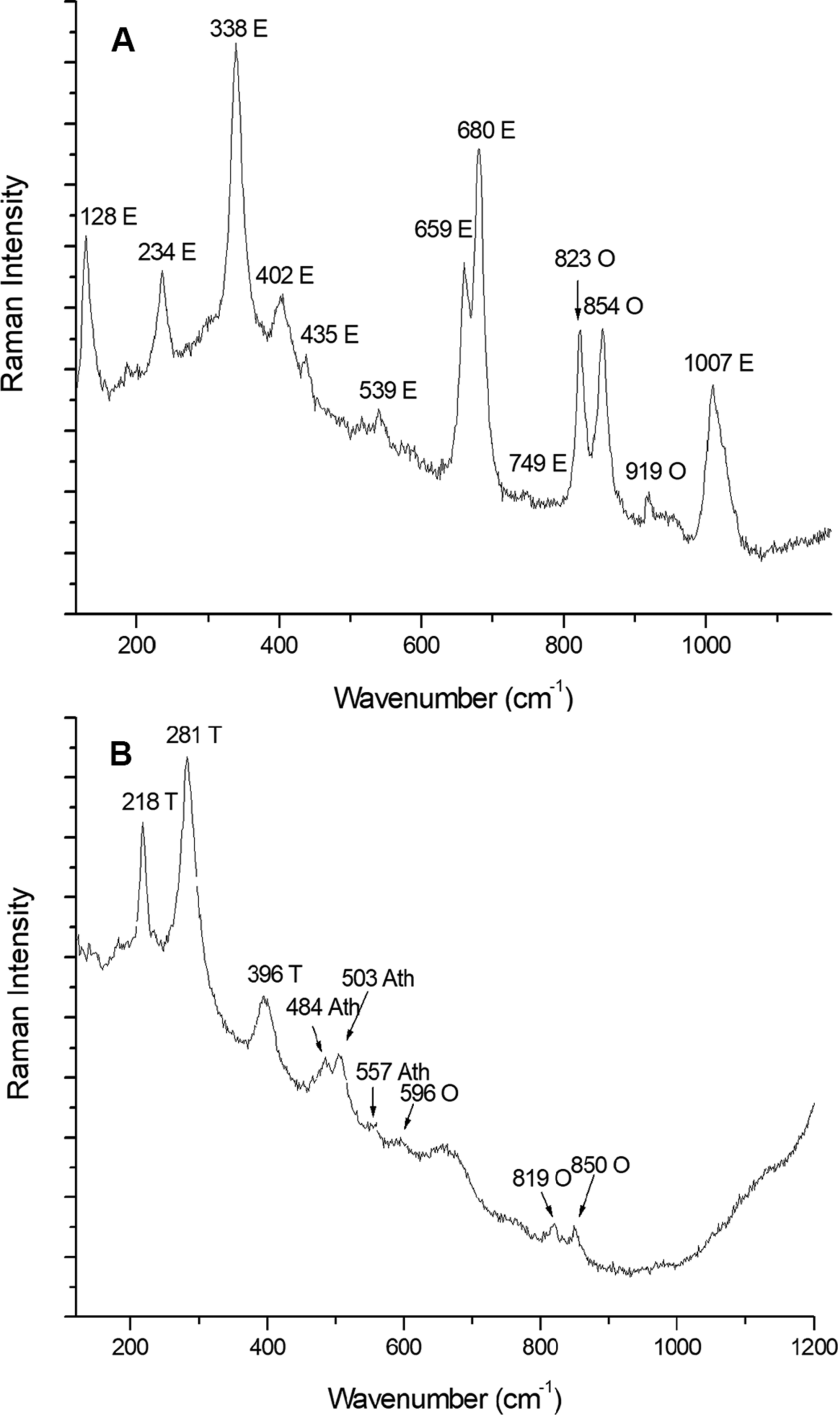
Primary mineral phases. (A) Raman spectrum of enstatite (E), together with olivine (O). Measurement parameters: Renishaw inVia Raman microspectrometer, 785 nm laser, 10% laser power, objective 50×, 4 s of exposure time, and 5 accumulations. (B) Raman spectrum of troilite (T), together with anorthite (Ath) and olivine (O). Measurement parameter: Renishaw inVia Raman microspectrometer, 785 nm laser, 10% laser power, objective 50×, 10 s of exposure time, and 20 accumulations.

Although there are 120 Raman-active modes for *Pbca* pyroxenes,^43^ the magnesium end member of the orthopyroxene mineral group, enstatite, was identified with its characteristic Raman bands at 128, 234, 338, 402, 435, 539, 659, 680, 749, and 1007 cm^–1^^44^ (Figure 1 A). It is well-known that enstatite usually consists of a part of the pyroxene’s composition. However, in this case of study, as can be observed in Figure 1 A, all the enstatite Raman bands found were identified together with the main olivine Raman bands. Taking into account that the Raman measurements were performed with the 50× objective and that the Raman spot size was of 1.3 μm, it can be said that enstatite and olivine appeared in the same micrometric grain. This fact led to think that enstatite is related to olivine.

The third major mineral present in the sample was olivine, which is the mineral group name given to the solid solution series between forsterite [Mg_2_SO_4_]^20^ and fayalite [Fe_2_SiO_4_].^20^ It is thought to be the dominant component in the Earth’s upper mantle^45^ and it normally appears in the general composition of stone and stony-iron meteorites.^46^ The Raman spectrum of the olivine has two strong bands, the first around 820 cm^–1^ and the second at 850 cm^–1^. According to Torre-Fdez et al.,^47,48^ if the Raman shift of both bands is known, the ratio of fayalite and forsterite in the olivine can be calculated using several equations that were critically checked in that work. Due to the discrepancies in the models, the same authors proposed other more sensitive models to calculate the magnesium content using the calibration shown in eqs 1 and 2, where *x* = % Mg.^47^ In order to obtain further information about the studied meteorite sample, the average of the wavenumber position of both Raman bands in all the olivine spectra was made with the aim of calculating the accurate composition of the olivines.
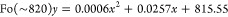
1
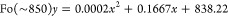
2

With eq 1 and with the position of the 820 cm^–1^ band as a value of *y*, the percentage in weight of forsterite in the sample was obtained. The position of the 850 cm^–1^ band was the *y* value of eq 2, and doing the average of the two values of *x*, the real forsterite percentage in the sample was calculated. In this way, the range of forsterite and fayalite in the NWA 11273 Lunar meteorite olivines goes from Fo_56_Fa_44_ to Fo_83_Fa_17_. Thus, these values indicate that the concentration of magnesium silicate is higher than that of the iron silicate, that is, there is more forsterite than fayalite. These data were consistent because the most mare basalt olivine grains contain only 20% Fa and very few olivines have more content of Fe than Mg.^49^

Apart from the three major mineral phases found, other lunar minerals were identified in minor quantities. One of these minerals is troilite (FeS), which was occasionally identified. Its main Raman peaks appeared at 218, 281, and 396 cm^–1^^50^ (Figure 1B). Troilite is often found in various extraterrestrial objects from meteorites to cosmic dust.^25^ Besides, it is the most common sulfur component found on the Moon because the low oxygen partial pressure in the lunar environment does not permit the formation of sulfate (SO_4_^2–^) minerals.^49^

**Figure 2:**
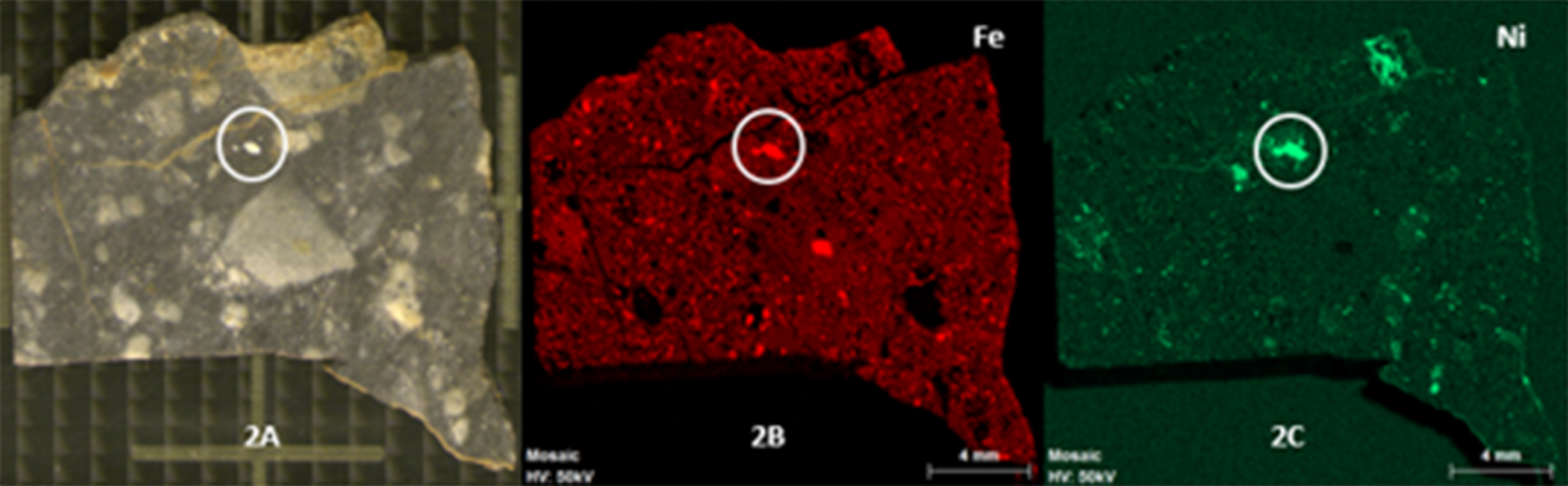
(A) X-ray fluorescence image of the B meteorite side, with the metallic area marked. (B) X-ray fluorescence image for iron and (C) nickel.

Furthermore, some areas of the meteorite showed a metallic appearance, which was distinguished by the naked eye (Figure 2A). In this way, those areas were studied by XRF and semiquantitative analyses were carried out obtaining a ratio of 93:7% of Fe/Ni in weight. This relation of iron and nickel indicated the presence of kamacite, the alpha (Fe,Ni) phase iron-nickel solution, that contains less than 6% of nickel.^51^ When the nickel content increases, the alloy changes to the gamma (Fe,Ni) phase, taenite, that contains between 25 and 50% nickel.^51^Figure 2B,C shows the distribution of iron and nickel in the meteorite surface. The intensity of color is directly correlated to the concentration of the element; thus, higher intensity means higher relative presence of each element and vice versa. The circled area in the figures corresponds to the main kamacite grain.

The studied sample is an achondrite and the metallized area is located in the middle of the sample. Hence, the kamacite presence may be due to the fusion between materials from the celestial body that impacted the Moon and the lunar regolith at the time of the meteorite’s formation, as a metallic mineral is not expected in this kind of meteorites.^52^

**Figure 3:**
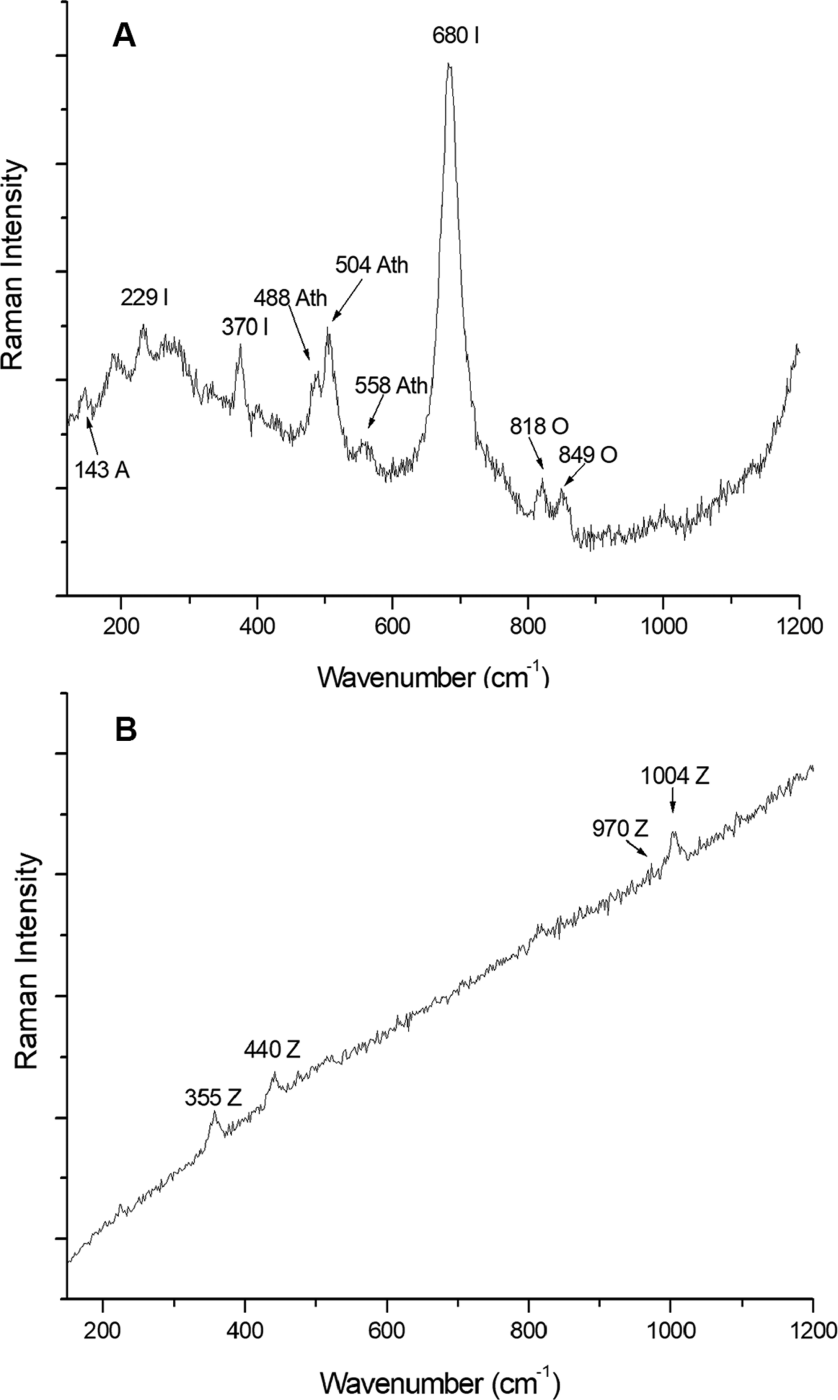
Primary mineral phases. (A) Raman spectrum of ilmenite (I), together with anatase (A), anorthite (Ath), and olivine (O). Measurement parameters: Renishaw inVia Raman microspectrometer, 532 nm laser, 5% laser power, objective 50×, 20 s of exposure time, and 2 accumulations. (B) Raman spectrum of the shocked zircon (Z). Measurement parameters: Renishaw inVia Raman microspectrometer, 532 nm laser, 5% laser power, objective 20×, 4 s of exposure time, and 1 accumulation.

Ilmenite [FeTiO_3_]^53^ and zircon [ZrSiO_4_]^54^ were also found in the NWA 11273 meteorite. Ilmenite is the most-abundant oxide mineral in lunar rocks and forms as much as 15–20% by volume of many Apollo 11 and 17 mare basalts.^49^ It was identified by its Raman bands at 229, 370, and 680 cm^–1^^55^ (Figure 3A).

Regarding zircon, which appears in less lunar rocks than ilmenite, it is worthy highlighting that it has the great capability to release the pressure it had been previously subjected to. This is because when zircon is subjected to a pressure higher than 20 GPa,^56^ its tetragonal structure (with space group *D*_4h_ 19 or *I*4_1_/*amd*; *a* = 6.607 Å; *c* = 5.981 Å)^56^ changes into the structural phase transition of the mineral reidite, the scheelite-structure phase (space group *I*4_1_/*a*; *a* = 4.734 Å; *c* = 10.51 Å).^56^

In this way, Gucsik et al.^56^ proposed that the main band of the zircon shifts its wavenumber by two units, in cm^–1^, for every 10 GPa it undergoes. Knowing that unshocked zircon has peaks at 202, 214, 225, 269, 355, 393, 439, 975, and 1008 cm^–1^,^57^ the Raman signals found at 355, 440, 970, and 1004 cm^–1^ (Figure 3B) could be used to extrapolate a rough estimation of the pressure reached during impact. In detail, as the main band of zircon suffered a displacement from 1008 to 1004 cm^–1^, the impact pressure suffered by the NWA 11273 Lunar meteorite was estimated to be around 20 GPa.^57^

**Figure 4:**
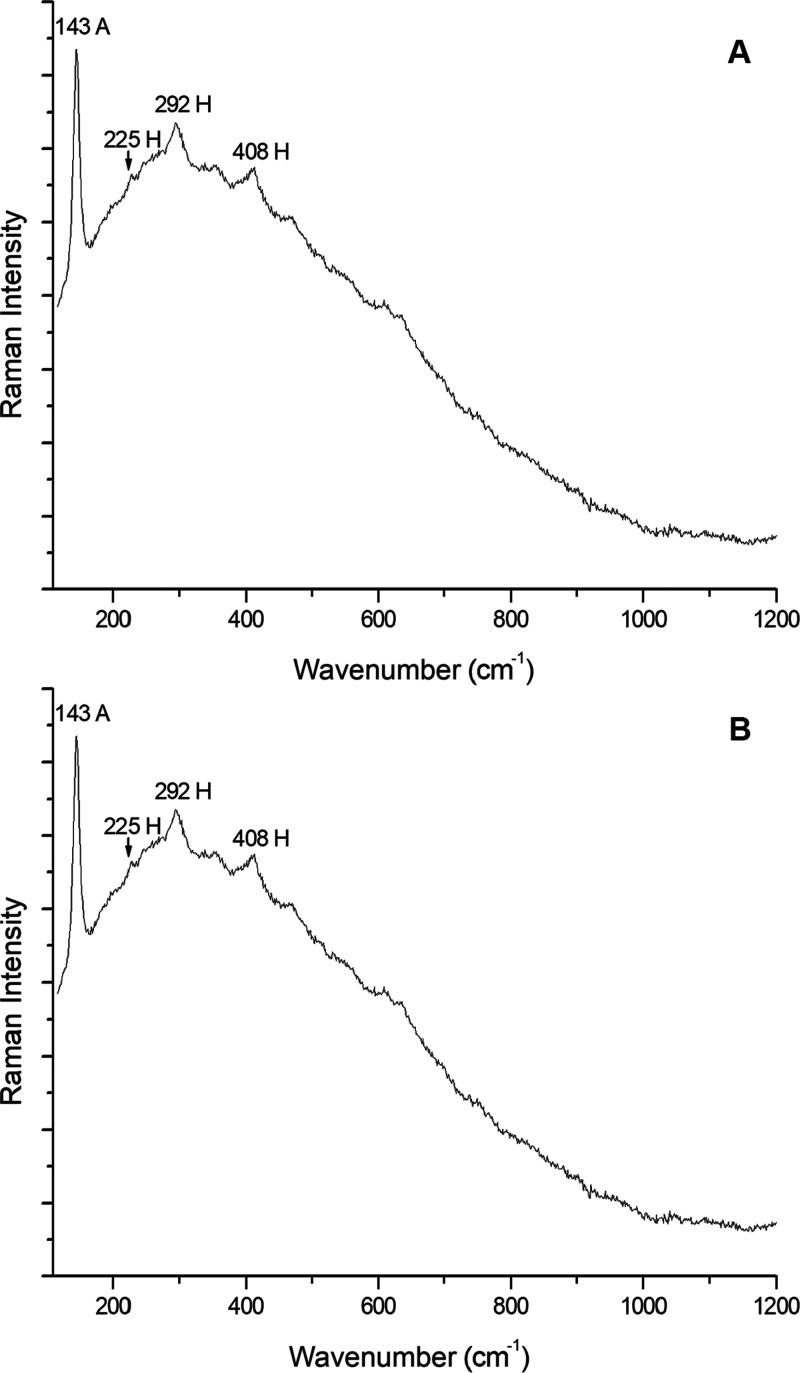
Secondary mineral phases. (A) Raman spectrum of quartz (Q) and hematite (H). Measurement parameters: Renishaw inVia Raman microspectrometer, 785 nm laser, 1% laser power, objective 50×, 25 s of exposure time, and 5 accumulations. (B) Raman spectrum of anatase (A), together with hematite (H). Measurement parameters: Renishaw inVia Raman microspectrometer, 785 nm laser, 1% laser power, objective 20×, 25 s of exposure time, and 5 accumulations.

Finally, three oxides were found in the sample: hematite [Fe_2_O_3_],^58^ quartz [SiO_2_],^59^ and anatase [TiO_2_].^60^ Hematite was found occasionally through point-by-point Raman analysis with bands at 225, 290, 405, 495, and 605 cm^–1^^61^ (Figure 4A). With regard to iron oxides, magnetite [Fe_3_O_4_]^62^ and hematite had been so far detected in lunar rocks and soils using a variety of techniques.^62^ However, sometimes, the appearance of iron oxides when measuring with Raman spectroscopy may be due to alteration of other mineral phases by the incidence of the laser at high powers. For example, other studies have checked that the observation of hematite by Raman spectroscopy can be caused by the oxidation of lunar ilmenite or the elemental iron during the analysis process in the presence of a terrestrial atmosphere.^63,64^ However, in this study, using different lasers at low intensity, the thermodecomposition of the sample was certainly avoided.

Quartz was found by its Raman bands at 205, 266, 352, and 464 cm^–1^^65^ (Figure 4A). Although it is one of the major compounds on terrestrial igneous, metamorphic, and sedimentary rocks, this mineral phase is rarely found on the Moon. In fact, this distinction is one of the major mineralogic differences between the Moon and the Earth. On the Moon, the silica minerals tend to concentrate with (a) chemical elements that are also rare on the Moon, such as the KREEP elements (potassium, REE, and phosphorous),^49^ or with (b) variable amounts of plagioclase, potassium feldspar, and pigeonite, mixture the so-called quartz monzodiorite.^66^ Taking this into account, the quartz found in the sample and studied did not appear accompanied with the mentioned element and mineral phases. In contrast, all quartz Raman spectra were always accompanied with hematite and olivine. Therefore, the presence of quartz and hematite seems to be produced as an alteration product of other primary Moon minerals. Both oxides come probably from the oxidation of natural olivines. It is well-known that this type of oxidation is a commonly recognized natural phenomenon caused first by a high-pressure event and then oxidation.^67^ In this way, the reaction occurs by an initial breakdown of the fayalite and forsterite components due to the high-pressure effect, then the oxidation of the fayalite component, and subsequent reaction with the forsterite one, to give magnetite and enstatite.^68^ The reactions are:
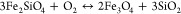

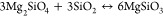


Due to the oxidation of the most susceptible olivines, hematite, quartz, and enstatite were found in the NWA 11273 Lunar meteorite. The detection of olivine remnants indicates that the alteration process was not complete.

The last oxide found was anatase, whose main Raman band appears at 143 cm^–1^^69^ (Figure 4B). It is one of the most-abundant mineral phases in the terrestrial nature, but anatase has not been found yet in the Moon, so its presence in the NWA 11273 Lunar meteorite could be associated with an alteration of other primary minerals, such as ilmenite, which is the most abundant oxide mineral on the Moon.^49^ Another titanium-bearing compound on the Moon is titanian chromite, but it is much less abundant than ilmenite. Anatase transforms irreversibly to the other titanium dioxide polymorph, rutile, at elevated temperatures higher than 800 °C.^70,71^ Therefore, the transformation of the titanium-bearing compound into anatase happened after the high-temperature events of the travel to the ground. Taking this into account, it is highly probable that the presence of anatase in the NWA 11273 meteorite may be due to an alteration related to the oxidation of the Fe(II) in ilmenite to form irreversibly hematite and anatase.^72^ In this work, we propose the following reaction, in which the iron of ilmenite is oxidized into hematite due to the oxidizing Earth atmosphere.
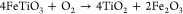


Apart from the previously discussed lunar minerals, other compounds that are not related with the Moon were found, for instance, calcite [CaCO_3_]^67^ and sulfate [SO_4_^2–^].^73^

**Figure 5:**
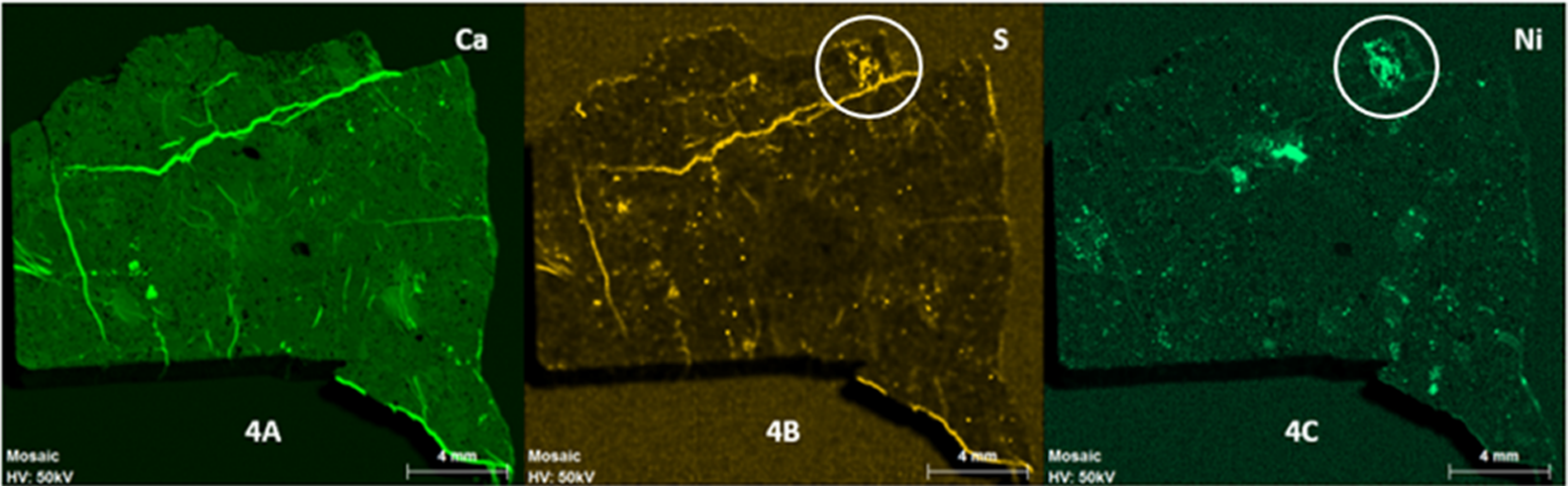
XRF images of (A) calcium, (B) sulfur, and (C) nickel.

**Figure 6:**
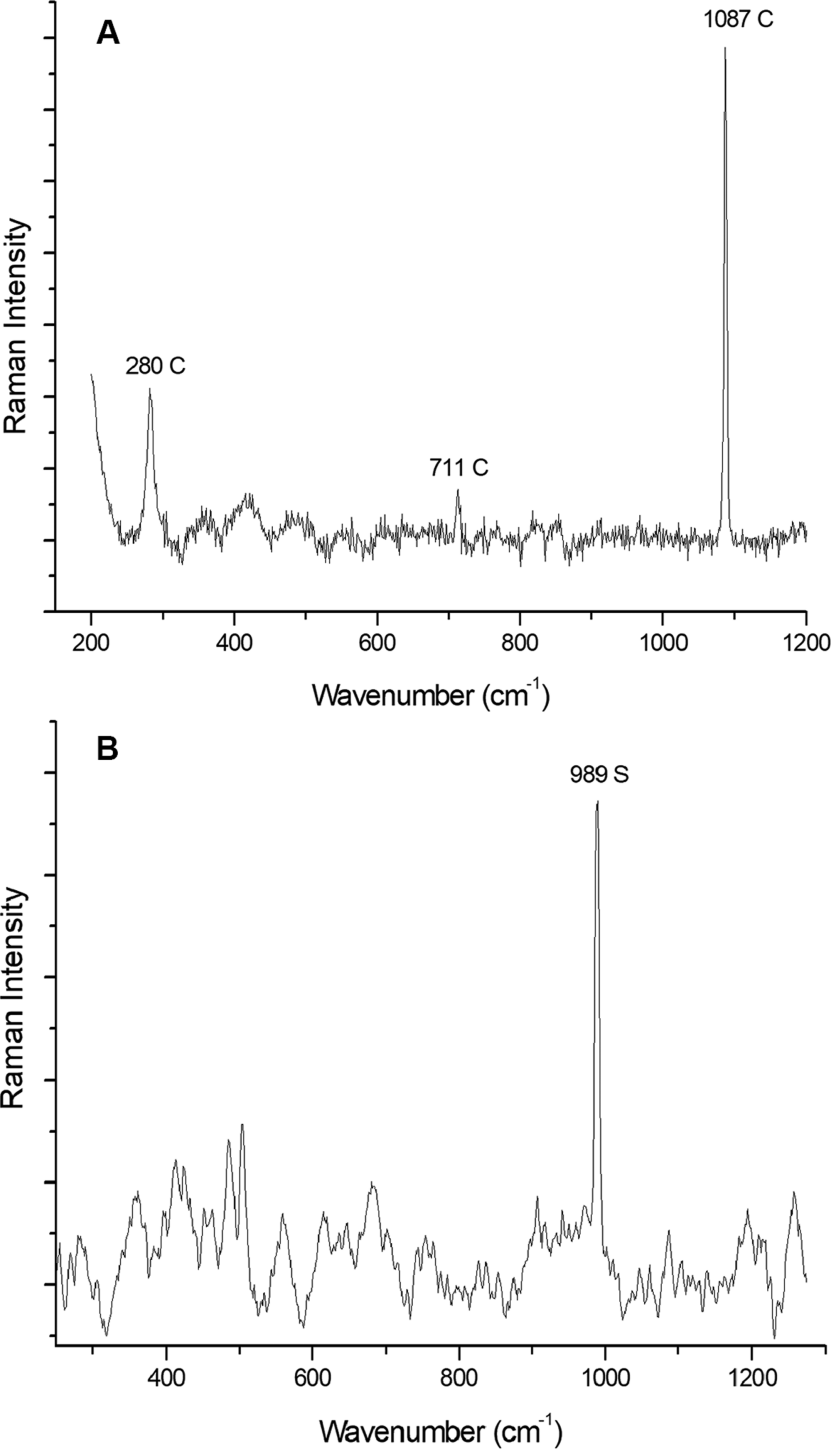
Terrestrial weathering mineral phases. (A) Raman spectrum of calcite (C). Measurement parameters: Renishaw inVia Raman microspectrometer, 785 nm laser, 1% laser power, objective 50×, 25 s of exposure time, and 5 accumulations. Spectrum treatment: baseline done. (B) Raman spectrum of sulfate (S). Measurement parameters: Renishaw inVia Raman microspectrometer, 785 nm laser, 1% laser power, objective 20×, 25 s of exposure time, and 5 accumulations. Spectrum treatment: baseline done.

The most-abundant unexpected mineral found in the sample was calcite, which was identified by combining XRF and Raman measurements. The XRF technique indicated that the calcium was distributed all along the meteorite (Figure 5A), but cracks showed higher concentration of this element than the rest of the surface. As Figure 5B shows, these fractures were also rich in sulfur, so, in order to know which mineral was present in the cracks, Raman analysis was carried out obtaining 280, 711, and 1087 cm^–1^ (Figure 6A), which correspond to those of calcite.^34^ It is possible that the high amount of calcite in the cracks masks the Raman signal of other compounds, such as gypsum [CaSO_4_·2H_2_O].^74^ As Figure 5A,B shows, sulfur and calcium coexist along the cracks. With regard to calcite, XRF did not detect carbon because of its low limit of detection. The most reliable hypothesis about the presence of calcite in the cracks of the meteorite is the precipitation of a solution saturated with the ions Ca^2+^ and HCO^3–^ at a pH higher than 8. These ions were transported in the water that entered in the meteorite through the cracks once on Earth. Then, water was evaporated, and the ions precipitated forming the mentioned calcium carbonate.

Figure 5C shows the elemental distribution of nickel along the side B of the meteorite. Apart from the kamacite areas, which are on the middle of the sample, there are other zones where nickel and sulfur coexist.

In order to find out the molecular composition of the region marked in which sulfur and nickel coexist, once again, Raman analyses were carried out. In this way, a unique and sharp band at 989 cm^–1^ (Figure 6B) was obtained. This band can be related to the sulfate anion.^73^ Unfortunately, it was impossible to detect any secondary band to identify the actual sulfate. However, due to the high spatial correlation of sulfur and nickel detected by XRF, it could be stated that the sulfate could correspond with retgersite [Ni(SO_4_)·6H_2_O]^34^ whose Raman bands are 206, 241, 462, and 986 cm^–1^.^34^ Until today, sulfates have not been reported in the Moon, so its presence may be due to terrestrial weathering.

**Figure 7:**
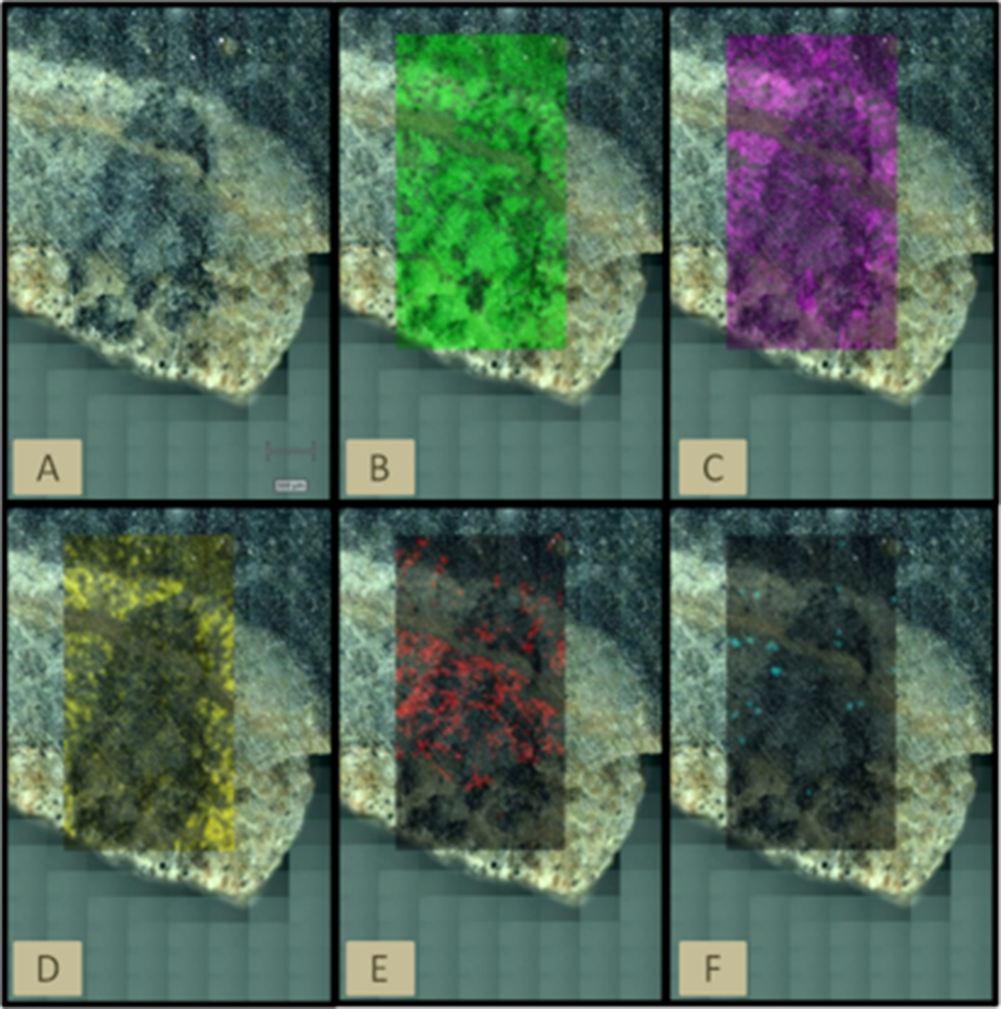
Raman images of the (A) sample area mapping, (B) anorthite, (C) pyroxene, (D) olivine, (E) calcite, and (F) sulfate obtained with the Renishaw inVia Raman microspectrometer.

Complementary to point-by-point analysis, big areas of the meteorite were analyzed by Raman imaging, which is an analytical method that allows investigating the spatial distribution and interaction of the molecular compounds. As such, Raman imaging was carried out in both sides of the meteorite sample. Figure 7A shows an optical image of a selected area where the main mineral phases of the meteorite were presented. Figure 7B shows the anorthite-rich area (in green), which was constructed after selecting the interval 498–512 cm^–1^ that contains the main Raman bands for anorthite. Figure 7C,D shows the spatial distribution of pyroxene (in purple, the area with a signal at the 665–685 cm^–1^ interval containing the common band of both types of pyroxenes) and olivine (in green, which was created using the 815–834 cm^–1^ interval that contains one of the main bands for olivine). By this means, it can be said that pyroxene and olivine were distributed along the matrix, such as anorthite. However, as can be seen, there is a grain that is not composed by olivine, as it is made of anorthite, pyroxene, and some calcite (Figure 7E). The Raman image of calcite (in red) was obtained using the interval 1082–1093 cm^–1^ that contains the main Raman band for calcite at 1084 cm^–1^. It has to be considered that due to the lack of flatness in the area analyzed (the crack is deeper than the rest of the region), the Raman signals of the compounds present in the crack do not appear in this Raman image. Other Raman measurements performed focused on the cracks showed the presence of calcite along them. The same happened with the sulfate present in the crack. However, Figure 7F shows the scarce distribution of sulfate (in blue) also out of the crack. This figure was constructed after selecting the 980–991 cm^–1^ where the main and unique band of the sulfate appears. As the sulfate may be associated with weathering processes, its distribution is not homogeneous and it appears occasionally.

## Conclusions

In this work, an analytical strategy based on the use of nondestructive spectroscopic techniques has been used to increase the knowledge about the mineralogical composition of the NWA 11273 Lunar meteorite. In detail, high-resolution XRF and Raman spectrometry were used, analytical techniques being onboard the NASA’s Perseverance Rover that is currently operating on Mars.

Using this strategy, it was possible to identify primary mineral phases of lunar origin, secondary mineral phases, formed through the alteration of the primary ones, and mineral phases associated with terrestrial weathering.

According to the Meteoritical Bulletin, this work confirms the presence of anorthite, olivine, pyroxene, troilite, and kamacite. In addition, thanks to the high resolution of the techniques used, two more primary minerals were identified: ilmenite and zircon.

Besides the primary minerals, hematite, quartz, enstatite, and anatase were detected as secondary minerals. In this work, it is proposed that hematite, quartz, and enstatite are minerals formed through the pressure alteration of olivine. While anatase is an alteration product of ilmenite. It was also possible to verify that the meteorite was subjected to high pressures since one of the primary minerals, zircon, is capable to reveal the pressure to which the sample was subjected to measuring the displacement of its Raman bands. Thus, in this work, we have estimated that the NWA 11273 Lunar meteorite was subjected to a pressure of about 20 GPa at the time of its formation. Hence, it can be confirmed that the proposed alteration processes may be due to the high pressures.

Although the point-by-point analyses gave us relevant information about the sample, it is important to highlight the great advantage of the imaging analyses. Both Raman and XRF imaging provide a visual distribution of the sample composition, which helped us to interpret the results. Thanks to imaging analysis, it was possible to identify the distribution of calcium and sulfur along the cracks, as well as nickel and sulfur in a cracked area in the upper part of the meteorite. These zones correspond to calcite and sulfate, possibly the hydrated nickel sulfate retgersite. Both mineral phases are products of terrestrial weathering and it is possible that they remained as precipitates after the entry of ion-rich water through the cracks.

As can be seen, by combining high-resolution nondestructive techniques such as XRF and Raman, a deeper mineralogical analysis can be carried out, being able to differentiate between primary, secondary, and terrestrial weathering mineral phases.

## Supporting Information Available

Figure of the sides A and B of the NWA 11273 Lunar Meteorite and all the mineral phases found in the sample with their molecular formula, their origin source, and the position of the main Raman bands detected (PDF)
