# Supplementary material for: Geochemical Characterization of the NWA 11273 Lunar Meteorite Using Nondestructive Analytical Techniques: Original, Shocked, and Alteration Mineral Phases
Source: ACS Earth Space Chem. 2021 May 27;5(6):1333–42. doi: 10.1021/acsearthspacechem.0c00329 (PMC9165041; doi:10.1021/acsearthspacechem.0c00329)
Supplement: Supplementary file 1 — sp0c00329_si_001.pdf [file sp0c00329_si_001.pdf]

SUPPORTING INFORMATION

Geochemical characterization of the NWA 11273 Lunar meteorite using non-destructive analytical techniques: original, shocked and alteration mineral phases

Jennifer Huidobro*, Julene Aramendia, Gorka Arana and Juan Manuel Madariaga

Analytical Chemistry Department, University of the Basque Country (UPV/EHU), Barrio Sarriena s/n, 48980 Leioa, Spain

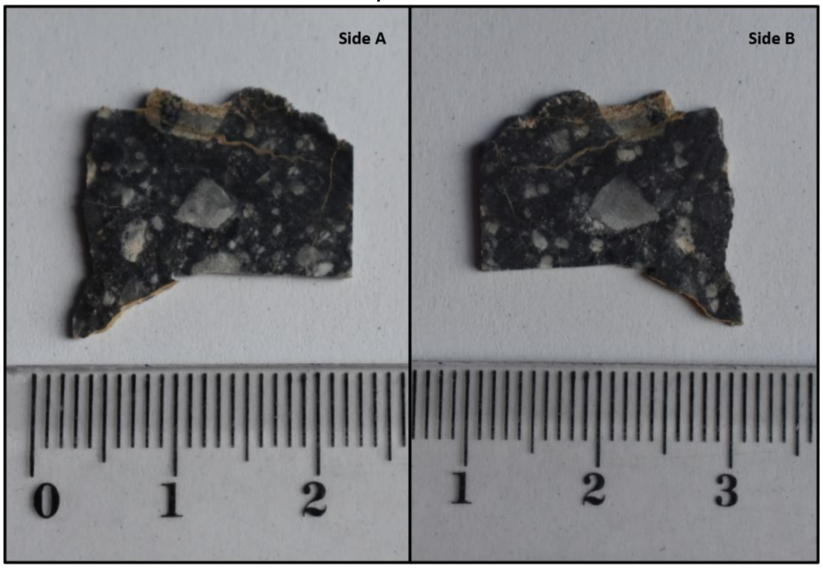

Figure 1. *Sides A and B of the NWA 11273 Lunar Meteorite.*

Table 1. *Mineral phases found in the NWA 11273 Lunar meteorite and their features* [a].

Mineral Phase	Molecular Formula	Source	Raman Bands (cm^{-1})
Anorthite	$CaAl_2Si_3O_8$	L	281 (m), 402 (m), 487 (sh s), 505 (vs), 560 (m), 681 (m), 766 (w)
Clinopyroxene	(Ca,Mg,Fe,Mn,Na,Li) (Al,Mg,Fe,Mn,Cr,Sc,Ti) $(Si,Al)_2O_6$	L	321 (m), 388 (s), 663 (s), 1010 (vs)
Orthopyroxene		L	335 (m), 660 (sh s), 678 (vs), 1004 (vs)
Enstatite	$MgSiO_3$	L/TW	128 (s), 234 (m),338 (vs), 402 (m), 435 (w), 539 (w), 659 (sh s), 680 (vs), 1007 (s)
Olivine	$(Fe,Mg)_2SiO_4$	L	421 (w), 600 (w), 673 (w), 820 (vs), 850 (vs), 958 (w)
Kamacite	Fe:Ni alloy	L/S	-
Troilite	FeS	L	218 (s), 281 (vs), 396 (m)
Ilmenite	$FeTiO_3$	L	229 (w), 370 (m), 682 (vs)
Zircon	$ZrSiO_4$	L	355 (s), 440 (s), 970 (w), 1004 (vs)
Hematite	Fe_2O_3	L/TW	225 (s), 290 (vs), 405 (s), 495 (w), 605 (m)
Quartz	SiO_2	L/TW	205 (w), 266 (w), 352 (m), 464 (s)
Calcite	$CaCO_3$	TW	153 (w), 280 (m), 711 (w), 1087 (vs)
Sulfate	SO_4^{2-}	TW	989 (m)
Anatase	TiO_2	TW	143 (s)

[a] Mineral phases found in the NWA 11273 Lunar meteorite, their source: Lunar (L), Space (S) or Terrestrial Weathering (TW) and their main Raman bands in the spectra obtained; v: very; s: strong; m: medium; w: weak; br: broad; sh: shoulder.
